# Anti-Cancer Activity of Resveratrol and Derivatives Produced by Grapevine Cell Suspensions in a 14 L Stirred Bioreactor

**DOI:** 10.3390/molecules22030474

**Published:** 2017-03-16

**Authors:** Laetitia Nivelle, Jane Hubert, Eric Courot, Philippe Jeandet, Aziz Aziz, Jean-Marc Nuzillard, Jean-Hugues Renault, Christophe Clément, Laurent Martiny, Dominique Delmas, Michel Tarpin

**Affiliations:** 1 Unité Matrice Extracellulaire et Dynamique Cellulaire, UMR CNRS 7369, SFR Cap-Santé FED 4231, UFR des Sciences Exactes et Naturelles, Université de Reims Champagne-Ardenne, BP 1039, 51687 Reims CEDEX 2, France; laetitia.nivelle@univ-reims.fr (L.N.); laurent.martiny@univ-reims.fr (L.M.); michel.tarpin@univ-reims.fr (M.T.); 2 Institut de Chimie Moléculaire de Reims, UMR CNRS 7312, SFR Cap-Santé FED 4231, UFR de Pharmacie, Université de Reims Champagne-Ardenne, 51687 Reims CEDEX 2, France; jane.hubert@univ-reims.fr (J.H.); jean-marc.nuzillard@univ-reims.fr (J.-M.N.); jh.renault@univ-reims.fr (J.-H.R.); 3 Unité de Recherche Vignes et Vins de Champagne EA 4707, SFR Condorcet FR CNRS 3417, UFR des Sciences Exactes et Naturelles, Université de Reims Champagne-Ardenne, BP 1039, 51687 Reims CEDEX 2, France; aziz.aziz@univ-reims.fr (A.A.); christophe.clement@univ-reims.fr (C.C.); 4 Centre de Recherche Inserm U866, Université de Bourgogne, 21000 Dijon, France; dominique.delmas@u-bourgogne.fr

**Keywords:** resveratrol, phytostilbenes, melanoma, fibroblasts, anticancer activity, bioreactor, *Vitis labrusca*

## Abstract

In the present study, resveratrol and various oligomeric derivatives were obtained from a 14 L bioreactor culture of elicited grapevine cell suspensions (*Vitis labrusca* L.). The crude ethyl acetate stilbene extract obtained from the culture medium was fractionated by centrifugal partition chromatography (CPC) using a gradient elution method and the major stilbenes contained in the fractions were subsequently identified by using a ^13^C-NMR-based dereplication procedure and further 2D NMR analyses including HSQC, HMBC, and COSY. Beside δ-viniferin (**2**), leachianol F (**4**) and G (**4′**), four stilbenes (resveratrol (**1**), ε-viniferin (**5**), pallidol (**3**) and a newly characterized dimer (**6**)) were recovered as pure compounds in sufficient amounts to allow assessment of their biological activity on the cell growth of three different cell lines, including two human skin malignant melanoma cancer cell lines (HT-144 and SKMEL-28) and a healthy human dermal fibroblast HDF line. Among the dimers obtained in this study, the newly characterized resveratrol dimer (**6**) has never been described in nature and its biological potential was evaluated here for the first time. ε-viniferin as well as dimer (**6**) showed IC_50_ values on the three tested cell lines lower than the ones exerted by resveratrol and pallidol. However, activities of the first two compounds were significantly decreased in the presence of fetal bovine serum although that of resveratrol and pallidol was not. The differential tumor activity exerted by resveratrol on healthy and cancer lines was also discussed.

## 1. Introduction

Resveratrol and its derivatives are secondary plant metabolites widely represented in nature and described as important defense molecules acting as phytoalexins in a few plants [1,2,3,4,5]. Phytostilbenes are implicated with human health benefits and possess many therapeutic actions such as anti-inflammatory [6], antioxidant [7], cardioprotective [8], anti-diabetic [9], anti-aging [10] and anticancer properties [11]. Studies dealing with the cancer-related activity of resveratrol are the most abundant, stating that this compound can act at multiple levels of the carcinogenesis process, from its initiation to invasive cancer [12]. The specific chemical structure of resveratrol, which is based on a hydroxylated stilbene skeleton, was reported to be directly involved in this activity. Works have already evidenced the role of the position of hydroxyphenyl groups on the stilbene skeleton on its antioxidant properties [13] as well as the importance of its stereochemistry on cell proliferation [14]. Study of the biological properties of resveratrol derivatives (hydroxylated, methylated, isoprenylated and oligomers) which are based on the same molecular structure, would thus allow the development of new anticancer agents. Interest in the activity of resveratrol derivatives is not recent [15,16] as previous works have already underlined the anticancer potential of piceatannol [17], a 3′-hydroxyresveratrol and pterostilbene [18], the 3,5-dimethyl-resveratrol.

Because of limitations for preparation of resveratrol and derivatives in large amounts by using conventional plant extraction procedures or chemical synthesis, the bio-production of stilbenes by plant cell suspensions in response to various elicitors such as cyclodextrins or methyl jasmonate [19,20,21], without recombinant genetic modification, represent a useful technique for their large-scale production [22,23].

We report here on the production in a 14 L stirred bioreactor of various phytostilbenes from grapevine cell suspensions of *Vitis labrusca* L. (Concord), their separation by centrifugal partition chromatography (CPC) to study their activity on the cell viability of two human skin malignant melanoma cancer cell lines (HT-144 and SKMEL-28) and a healthy human dermal fibroblast HDF line.

## 2. Results and Discussion

### 2.1. Growth and Bioproduction of Phytostilbenes in the 14 L Bioreactor

**Figure 1:**
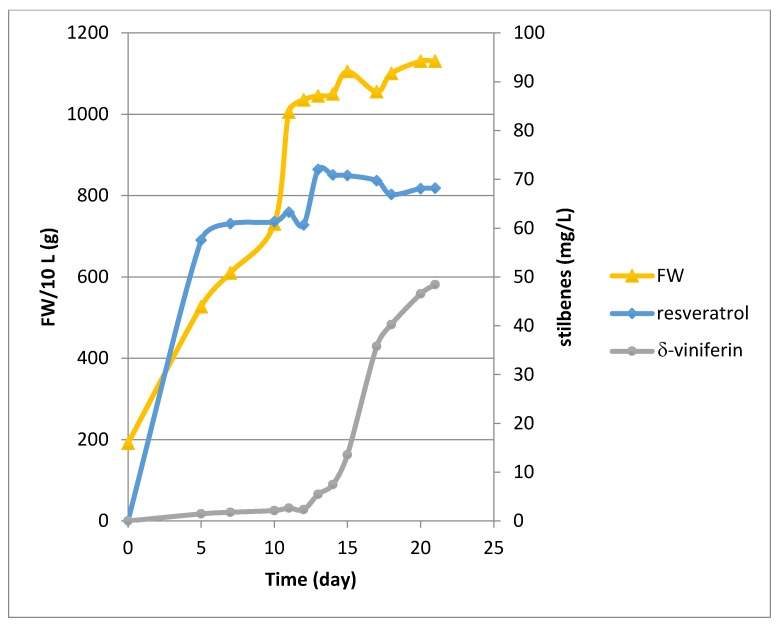
Growth kinetic and stilbene production (resveratrol, δ-viniferin) in the 14 L Bioflo 3000 bioreactor. Concord cells were cultured with CDR from Day 0 to Day 21 and MeJA was added at Day 11.

Bioreactor culture experiments were conducted over a period of 21 days in the presence of β-cyclodextrins (CDR). Methyl jasmonate (MeJA) was only added at Day 11 of the culture. Both cyclodextrins and MeJA are known to be elicitors of stilbene production in grapevine cell suspensions [19,20,21,22,23]. Cell growth showed an exponential phase (Figure 1) followed by a stationary phase upon addition of MeJA. The growth rate, determined between Day 0 and Day 11, gave a μ value of 0.15 day^−1^ corresponding to a Td of 4.59 days. As currently observed with our Concord cell line, no resveratrol production occurred without elicitation (Day 0). In presence of the CDR alone, resveratrol reached about 60 mg/L during the exponential phase followed by a peak at 72 mg/L at Day 13, two days after the addition of MeJA. Then its level remained almost constant until the end of the culture with a value of 68.1 mg/L (Figure 1). The production of δ-viniferin was quite low in presence of the CDR alone, its biosynthesis being activated after MeJA addition, with a production of 48.4 mg/L at Day 21 (Figure 1). Only resveratrol and δ-viniferin were quantified by UPLC, but several other peaks occurred on the chromatograms corresponding to resveratrol derivatives, the biosynthesis of which was only activated upon addition of MeJA.

Cell suspensions were dedicated to produce enough resveratrol and derivatives to allow their purification and biological evaluation. Resveratrol bioproduction by grapevine cell cultures is now well described and use of a combined cyclodextrin-MeJA elicitation can lead to stilbene production in the g/L range [20,21,22,23]. Methyl-β-cyclodextrins have been described as the most efficient class of cyclodextrins, but the quantity needed in the culture medium is quite important (65 g/L corresponding to 50 mM) [21] and their price is very expensive, especially for their use in bioreactors of several liters. Thus the less expensive native β-cyclodextrins were selected in this work in order to limit the cost of the culture.

### 2.2. CPC Separation and NMR Identification of Resveratrol and Its Bio-Produced Derivatives

In a single run of 160 min, 1.5 g of the crude ethyl acetate stilbene extract obtained from the culture medium was fractionated by centrifugal partition chromatography (CPC) using a gradient elution method. CPC is a solid support-free separation technique involving the distribution and the transfer of solutes between at least two immiscible liquid phases according to their distribution coefficient. The CPC column used in this work, with a total capacity of 303 mL and only 231 partition cells (FCPE300^®^), offered the possibility to collect fractions in sufficient quantities for further chemical analyses and biological assays. The CPC fractions eluted with the organic mobile phases over the gradient in the ascending mode contained all bio-produced stilbene derivatives and represented 73% of the injected mass, while the most hydrophilic compounds of the extract mainly including residual cyclodextrins and culture medium nutrients were well-retained inside the CPC column.

The major stilbenes contained in the CPC fractions were firstly identified by using a ^13^C-NMR-based dereplication procedure [24,25]. All CPC fractions from F1 to F11 were analyzed by ^13^C-NMR spectroscopy. Automatic peak picking and alignment of ^13^C-NMR signals across spectra of the fraction series resulted in a table with 11 columns (one per fraction) and 221 rows (one per chemical shift bin containing at least one ^13^C-NMR signal in at least one fraction). This table was submitted to Hierarchical Clustering Analysis (HCA) on the rows. As a result, statistical correlations between ^13^C-NMR resonances belonging to a single structure within the fraction series were visualized as “chemical shift clusters” in front of the corresponding dendrograms.

**Figure 2:**
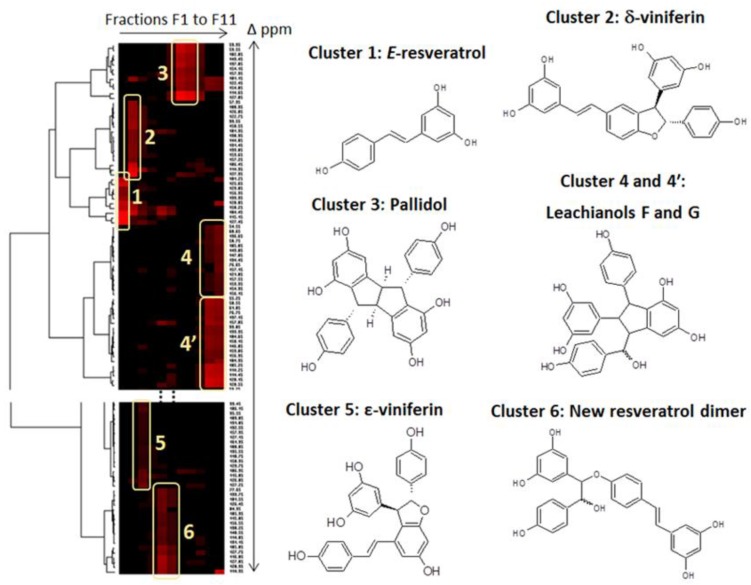
^13^C-NMR chemical shift clusters obtained by applying Hierarchical Clustering Analysis on the CPC fractions of the crude ethyl acetate extract obtained from the culture medium.

In the HCA correlation map presented in Figure 2, several well-defined clusters were intensely colored in red. Cluster 1 corresponded to an intense cluster of ten ^13^C-NMR chemical shifts. After entering these chemical shifts into a database containing ^13^C-NMR data of natural compounds, including 101 known stilbene structures from different plant genera, the structure of *trans*-resveratrol (**1**) was proposed as the first hit out of 60 proposals. This structure was confirmed by checking all the chemical shifts of *trans*-resveratrol in NMR spectra of fractions F1 in which the intensity of cluster 1 was predominant. By using the same method, clusters 2, 3, and 5 were identified as δ-viniferin or maximol A (**2**), pallidol (**3**), and ε-viniferin (**5**). Clusters 4 and 4′ corresponded to leachianol F (**4**) and leachianol G (**4′**), respectively, which were both present as a diastereoisomer mixture in fractions F10 and F11. Cluster 6 did not match with any known stilbene molecular structure stored in our database. Further 2D NMR analyses including HSQC, HMBC, and COSY were performed on fractions F5 and F6 containing the signals of cluster 6, leading to the identification of a new resveratrol dimer (**6**) which has never been described previously. To our knowledge, only one report has described the chemical synthesis of a methoxy-substituted derivative of this compound together with a series of other hydroxystilbene analogs in order to investigate the catalytic action of a laccase from *Trametes pubescens* on the dimerization of hydroxystilbenes [26].

In sum, 350 mg of *trans*-resveratrol (**1**), 42 mg of δ-viniferin (**2**), 206 mg of Pallidol (**3**), 187 mg of ε-viniferin (**5**) and 41 mg of the new resveratrol dimer (**6**) were obtained with purity values greater than 93%. These recovered quantities were sufficient to perform biological assays. To our knowledge, this is the first description of a 14L bioreactor able to produce such a diversity of resveratrol derivatives, especially using β-cyclodextrins.

### 2.3. Effects of Resveratrol and Bio-Produced Stilbenes on Cancer Lines and Healthy Human Dermal Fibroblasts

#### 2.3.1. Comparison of the Inhibition Activity of Bio-Produced and Synthetic Resveratrol on Cell Viability

**Figure 3:**
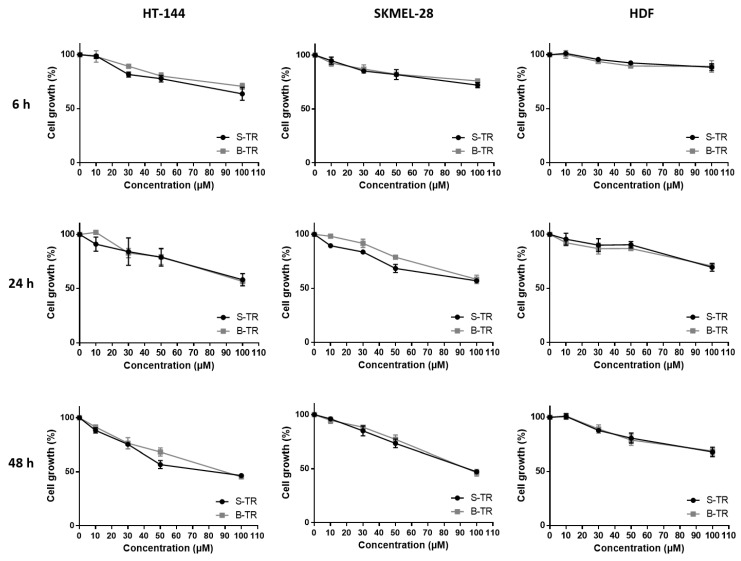
Cell viability inhibition of two different sources of *trans*-resveratrol. HT-144, SKMEL-28 and HDF cells were treated with various concentrations (10–100 μM) of synthetic *trans*-resveratrol (S-TR) and bio-produced *trans*-resveratrol (B-TR) for 6, 24 and 48 h. Cell viability was determined by the MTT assay. The final volume of ethanol was 0.01% and 0.1% for 10 and 100 μM concentrations, respectively. Data are expressed as the means ± SD of 3 independent experiments. Each experiment was performed in triplicate

The cell viability inhibition of two human skin malignant melanoma cancer cell lines (HT-144 and SKMEL-28) and a healthy human dermal fibroblast HDF line of the bio-produced resveratrol was compared to the one exerted by synthetic resveratrol as determined by the MTT assay (Figure 3). Results showed some similarities between synthetic and bio-produced resveratrol for the three used cell types. A dose-dependent decrease in cell growth was observed which well correlates with cell viability inhibition. Moreover, the slope of the curves increased over time, showing that the cell growth decrease is also time-dependent. Interestingly, diminution in the cell viability was less pronounced whatever the source of resveratrol on the healthy primary culture compared to the two melanoma cell lines.

As the purity of bio-produced compounds is generally lower compared to synthetic ones, this could interfere with their biological properties. However, we clearly showed here that whatever the origin of resveratrol, both compounds exert the same inhibiting effect on cell viability. Similar data have already been published regarding the biological activity of synthetic resveratrol and naturally occurring resveratrol on colon cancer cell (SW480) proliferation [27] and the metabolism of the precursor protein of amyloid [28]. Our results also revealed a tumor-specificity of resveratrol, that is, the cell viability inhibition exerted by this compound was found to be lower on the healthy human dermal fibroblasts, HDF compared to the two cancer cell lines, HT-144 and SKMEL-28. This confirms a previous work demonstrating that resveratrol employed at similar doses on healthy or cancer cells has no effects on the former [29]. The differential activity of resveratrol on healthy and cancer cells would avoid the heavy side effects induced by conventional cancer therapies which are unable of specifically targeting normal cells. Resveratrol may also display protective effects on normal cells namely in anticancer therapies involving both chemotherapeutic agents [30] and radiotherapy [31]. This difference in resveratrol activity which depends on the cell type has already been reported in case of its oligomeric derivatives. Chowdhury et al. studied the tumor-specificity of phytostilbenes and flavonoids, and highlighted a specificity of action of three oligomers in terms of cell viability decrease on cancer cells [32]. Another work has reported α-viniferin and *trans*-miyabenol C, two resveratrol trimers to have a higher inhibiting activity of the cell viability of three human colorectal carcinoma cell lines (HCT-116, HT-29 and Caco-2) than that of a healthy colorectal cell line (CCD-18Co) [33]. In the same way, gnetin H, another resveratrol trimer, was found to display lower IC_50_ values among four cancer cell lines (A549 lung carcinoma cells, BT20 and MCF7 breast carcinoma cells and U2OS osteosarcoma cells) as compared to healthy cell lines (HPL1A pulmonary epithelium and HMEC breast epithelium) [34].

#### 2.3.2. Inhibition of Cell Viability by Bio-Produced Resveratrol Oligomers

**Figure 4:**
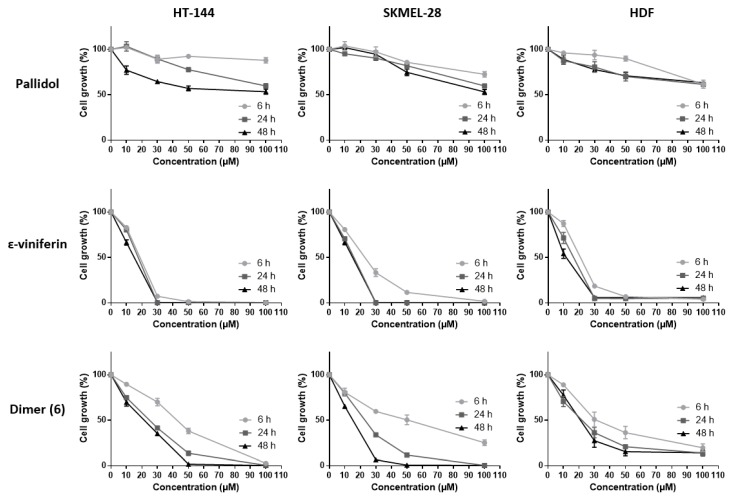
Cell viability inhibition of bio-produced resveratrol oligomers. HT-144, SKMEL-28 and HDF cells were treated with various concentrations (10–100 μM) of pallidol, ε-viniferin and dimer (**6**), three bio-produced reveratrol oligomers, for 6, 24 and 48 h. Cell viability was determined by the MTT assay. The final volume of ethanol was 0.01% and 0.1% for 10 and 100 μM concentrations, respectively. Data are expressed as the means ± SD of 3 independent experiments. Each experiment was performed in triplicate.

The inhibitory activities of the various resveratrol oligomeric compounds recovered from the grapevine cell suspensions (pallidol, ε-viniferin and the newly identified dimer (**6**)) on the cell viability of the three tested cell lines were evaluated using the MTT assay (Figure 4). Among the oligomeric resveratrol derivatives, pallidol displayed an inhibition of cancer line viability similar to that of resveratrol. However, this compound showed a less marked differential activity regarding healthy cell lines. The dehydrodimer ε-viniferin as well as the dimer (**6**) also showed a significant cell viability inhibition on the three tested cell lines, this activity being considerably higher than the one exerted by resveratrol.

A number of studies have already brought to the fore the anticancer potential of various stilbene oligomers originating from plants. Some of them have already shown inhibiting effects on cell viability of cancer cell lines: pallidol, for example, on human colorectal carcinoma cell lines (HCT-116, HT-29 and Caco-2) [33]; ε-viniferin on murine leukemia cell lines (P-388) [35], human oral squamous carcinoma cell lines (HSC-2, HSC-3), human sub-mandibular gland carcinoma cell lines (HSG), human promyelocytic leukemia cells (HL-60) [32], lymphoid and myeloid cell lines (U266, RPMI-8226, U937, K562, Jurkat) [36]. Other oligomeric stilbenes such as maximol A (δ-viniferin) also exert inhibiting effects on cell viability among human leukemia cells (HL-60), mammary cancer lines (MCF-7) and lung carcinoma cells (A-549) [37]. Hopeaphenol, a tetrameric stilbene, displays a similar activity against human hepatocarcinoma cell lines (HEPG2) [38] and murine leukemia lines (P-388) [39].

#### 2.3.3. Effect of Fetal Bovine Serum on the Biological Activity of the Bio-Produced Stilbenes

**Figure 5:**
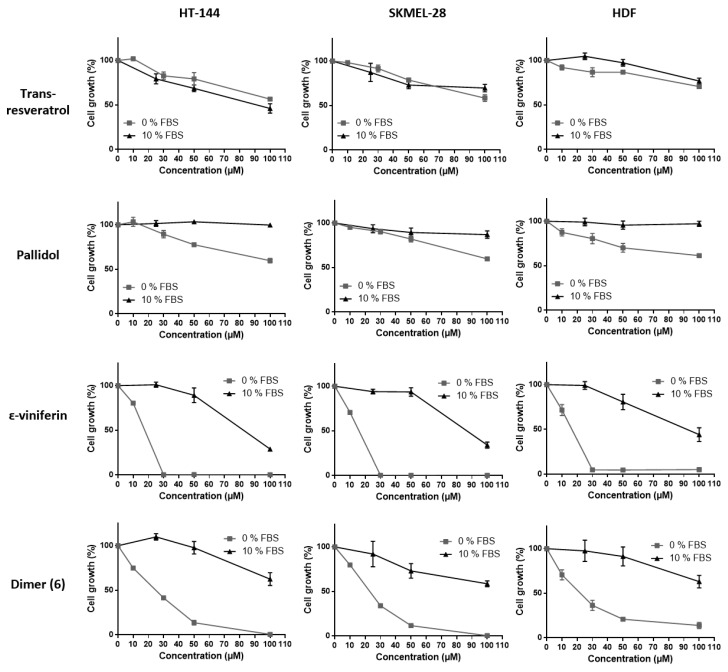
Cell viability inhibition of bio-produced stilbenes (resveratrol and the three dimers) with or without FBS. HT-144, SKMEL-28 and HDF cells were treated with various concentrations (10–100 μM) of bio-produced stilbenes for 24 h with FBS (10% FBS) or not (0% FBS). Cell viability was determined by the MTT assay. The final volume of ethanol was 0.01% and 0.1% for 10 and 100 μM concentrations, respectively. Data are expressed as the means ± SD of 3 independent experiments. Each experiment was performed in triplicate.

To study whether the fetal bovine serum (FBS) exerts any effect on stilbene activity, their capacities to inhibit cell viability were evaluated in the presence or absence of FBS on the three cell lines using the MTT assay (Figure 5). It is indeed well-known that polyphenols, namely resveratrol, strongly interact with serum proteins, this interaction resulting in a decrease of their intrinsic action. Inhibition of the cell viability displayed by ε-viniferin and dimer (**6**) was significantly decreased in the presence of FBS and to a greater extent than that of pallidol and resveratrol on the three tested cell lines. This is confirmed by the IC_50_ values obtained for each compound as shown in Table 1. Here again, ε-viniferin as well as dimer (**6**) showed IC_50_ values on the three tested cell lines considerably lower than the ones exerted by resveratrol and pallidol.

Though FBS strongly affects the capacity of stilbene oligomers to inhibit the viability of healthy and tumor cells, resveratrol efficiency on cancer cells was not decreased in the presence of FBS. This is so far not surprising as this compound can bind to serum proteins thus facilitating its transport to the cell [40]. It seems that interactions taking place between resveratrol oligomers and serum proteins, have a decreasing effect on their biological activity on cells. Such interactions could modify the protein conformation resulting in changes in their function as transporters in the cell. It has indeed been shown that the binding of polyphenols to the serum proteins can induce significant modifications of their secondary structures [41].

## 3. Materials and Methods

### 3.1. Chemicals, Reagents and Materials

Methanol (MeOH), Methyl-*ter*-butyl ether (M*t*BE), ethyl acetate (EtOAc), acetonitrile (CH_3_CN) and *n*-heptane (Hept) were purchased from Carlo Erba Reactifs SDS (Val de Reuil, France). Deuterated methanol (methanol-*d*_4_) was purchased from Sigma-Aldrich (Saint-Quentin, France). Deionized water (H_2_O) was used to prepare all aqueous solutions. Synthetic resveratrol was purchased from Sigma-Aldrich (Saint-Quentin, France).

### 3.2. Bioreactor Culture and Elicitation Process

Three flasks (1 L each) containing 400 mL of Concord (*Vitis labrusca*) cell suspension were cultured for one week in B5 medium [42], supplemented with 3% sucrose, α-naphtalene acetic acid (0.1 mg/L) and kinetin (0.2 mg/L), in darkness at 23 °C on a rotary shaker (Infors Orbitron, Massy, France) at 110 rpm. After 7 days of culture, the flasks were pooled in a 2 L flask and stirred before filtration on a 500 μm mesh sieve. The 570 g of filtered cells obtained were resuspended in a 2 L flask containing 1 L of the same medium complemented with 13 mM of β-cyclodextrin Kleptose^®^ (CDR) (Roquette, Lestrem, France) (B5C medium). The cells were then transferred using a peristaltic pump into a 14 L tank of a stirred bioreactor Bioflo 3000 (New Brunswick Scientific, New York, USA) containing 5 L of B5C medium. The agitation (2 marine turbines) was set to 50 rpm and the aeration rate was maintained at 0.025 vvm. After 4 days of culture, 2 L of B5C medium were added by pumping, followed by 1 L at day 7 and 1 L at day 10 in order to reach a 10 L final volume in the bioreactor. At day 11, 20 mL of a 400 mM MeJA solution in ethanol was added in the culture using a 20 mL sterile syringe to get a final MeJA concentration of 0.8 mM.

Growth and resveratrol production were monitored during 21 days by analysis of 25 mL samples obtained by the sample tool of the bioreactor. The samples were filtered under reduced pressure in order to measure the fresh weight and 20 mL of the filtered medium were washed with 20 mL of EtOAc for resveratrol analysis. The extracts were evaporated using a rotavapor (Laborota 4000-Efficient, Heidolph with PC3001 VARIO vacuum pump,Vacuubrand, Schwabach, Germany) and then re-suspended in 1 mL MeOH. Resveratrol quantification was realized by UPLC analyses as previously described [25] (see also Section 3.5.).

### 3.3. Extraction of Total Stilbenes from the Culture Medium and CPC Fractionation

The whole culture medium (9 L) was filtered and stored at −20 °C until use. A crude stilbene extract (3.1 g) was obtained from 5 L of the filtered culture medium by performing 3 successive extractions with ethyl acetate (3 × 2 L) in a separatory funnel followed by solvent elimination under vacuum at 40 °C.

Centrifugal Partition Chromatography (CPC) was carried out on a lab-scale FCPE300^®^ column of 303 mL capacity (Rousselet Robatel Kromaton, Annonay, France) containing 7 circular partition disks, engraved with a total of 231 oval partition twin-cells(~1 mL per twin-cell) and connected to a KNAUER Preparative 1800 V7115 pump (Berlin, Germany). The system was coupled to a UVD 170S detector set at 210, 254, 280, and 366 nm (Dionex, Sunnivale, CA, USA). Fractions were collected by a Pharmacia Superfrac collector (Uppsala, Sweden).

The crude stilbene extract was fractionated by using a gradient elution method as described previously [25]. Briefly, two biphasic solvent systems were prepared independently by mixing *n*-heptane, EtOAc, MeOH, and water in the proportions 5/6/5/6 (*v*/*v*) for system 1 and 2/5/2/5 (*v*/*v*) for system 2. These two systems were selected in order to increase the polarity of the mobile phase gradually during the gradient from system 1 to system 2 while ensuring the biphasic character of the overall system with MeOH and water as the major constituents in the lower phase. The sample solution was prepared by directly dissolving the crude stilbene extract (1.5 g) in 30 mL of a mixture of upper and lower phases of system 1 (50/50, *v*/*v*). The CPC column was filled with the lower aqueous phase of system 1 at 500 rpm. After loading the sample solution into the column through a 35 mL sample loop, the rotation speed was adjusted to 1200 rpm and the flow rate was gradually increased from 0 to 20 mL/min in 3 min and maintained at 20 mL/min over the whole experiment. The gradient was performed in the ascending mode by pumping 100% of the upper phase of system 1 for 5 min, and then this phase was decreased from 100% to 80% in 40 min, from 80% to 20% in 60 min, and from 20% to 0% in 20 min. The upper phase of system 2 was maintained at 100% for 35 min. Finally, the column was extruded by switching from the ascending to the descending mode, while still pumping 100% of the upper phase of system 2 at 20 mL/min. Experiments were conducted at room temperature. Fractions were collected every minute and spotted on Merck TLC plates coated with silica gel 60 F254 and developed with toluene/ethyl acetate/acetic acid/formic acid (30:70:11:11, *v*/*v*/*v*). After detection at UV254 and UV366, the plates were sprayed with vanillin–sulfuric acid and heated to 100 °C for 5 min. Fractions were then pooled according to their TLC profile similarities and evaporated under vacuum. As a result, 11 fractions were obtained.

### 3.4. NMR Analyses of the CPC Fractions and Identification of the Major Bio-Produced Stilbenes

An aliquot of each fraction from F1 to F11 (maximum of 20 mg) was dissolved in 600 μL of methanol-*d*_4_. NMR analyses were performed at 298 K on a Bruker Avance AVIII-600 spectrometer (Karlsruhe, Germany) equipped with a cryoprobe optimized for ^1^H detection and with cooled ^1^H, ^13^C and 2D coils and preamplifiers. ^13^C-NMR spectra were acquired at 150.91 MHz. A standard zgpg pulse sequence was used with an acquisition time of 0.909 s and a relaxation delay of 3 s. For each sample, 1024 scans were co-added to obtain a satisfactory signal-to-noise ratio. The spectral width was 238.9070 ppm and the receiver gain was set to the highest possible value. A 1 Hz line broadening filter was applied to each FID prior to Fourier transformation. The spectra were manually phased and baseline corrected using the TOPSPIN 3.2 software (Bruker) and calibrated on the central resonance (δ 49.1 ppm) of methanol-*d*_4_. A minimum intensity threshold of 0.3% (relative to the most intense signal of each spectrum) was then used to automatically collect all positive ^13^C-NMR signals while avoiding potential noise artifacts. Each peak list was then converted into a text file. Absolute intensities of the collected peaks in the fraction series were aligned by using an in-house algorithm written in the python language. The principle was to divide the ^13^C spectral width (from 0 to 200 ppm) into regular bins, i.e. chemical shift intervals (∆δ = 0.2 ppm), and to associate the absolute intensity of each ^13^C peak to the corresponding bin. The bins for which no signal was detected in any fraction were removed from the bin list. The resulting table was imported into the PermutMatrix version 1.9.3 software (LIRMM, Montpellier, France) for clustering analysis on peak intensity values. The classification was performed on the rows only, i.e. on the chemical shift bins. The Euclidian distance was used to measure the proximity between samples and the Ward’s method was used to agglomerate the data. The resulting ^13^C chemical shift clusters were visualized as dendrograms on a two-dimensional map (Figure 2). The higher the intensity of ^13^C-NMR peaks, the brighter the color on the map.

In parallel, a literature survey was performed to obtain names and structures for a maximum of stilbenes already described in the literature. In total, 101 compounds were found, mostly including monomers, dimers, and tetramers of resveratrol. These metabolites were added to a locally built ^13^C-NMR chemical shift database (ACD/NMR Workbook Suite 2012 software, ACD/Labs, Ontario, Canada) comprising the chemical shifts and structures of other natural products (≈1950 in November 2016). Structures were drawn with ACD/Labs ChemSketch. A ^13^C-NMR predicted spectrum was calculated with the ACD/Labs CNMR Predictor software and the resulting ^13^C-NMR chemical shifts were supplied to the database. For metabolite identification, each ^13^C-NMR chemical shift cluster obtained from HCA was manually submitted to the structure search engine of the database management software. A ^13^C-NMR chemical shift tolerance of ±2 ppm was used. Additional 2D NMR experiments (HSQC, HMBC, and COSY) were performed on the same Bruker Avance AVIII-600 spectrometer, by using standard Bruker pulse programs (Bruker, Karlsruhe, Germany) in order to confirm the structures of some minor compounds.

### 3.5. UPLC Analyses

UPLC analyses were done using an Acquity UPLC (Waters, Guyancourt, France) system, with a C18 Cortecs^®^ (Waters) column (2.1 × 100 mm, particles: 1.6 μm, pore size: 90 Å), maintained at 30 °C. For the separation, a gradient at 0.5 mL/min starting from 90% H_2_O + 0.1% formic acid (A) and 10% acetonitrile + 0.1% formic acid (B) to 40% A and 60% B, followed by 5 min rinsing was used. Stilbene fluorescence was measured with an Aqcuity fluorometer (Waters) with an excitation wavelength of 330 nm and an emission wavelength at 375 nm [25].

### 3.6. Biological Tests

#### 3.6.1. Cell Cultures

Three cell types were studied. The HT-144 and SKMEL-28 cell lines derived from human melanoma and were obtained from the American Tissue Culture Collection (ATCC). They were grown in Mc Coy’s 5a modified and minimal essential medium (MEM) respectively, supplemented with 10% (*v*/*v*) fetal bovine serum (FBS) and 1% (*v*/*v*) antibiotic (penicillin, streptomycin) at 37 °C in a humidified atmosphere of 5% CO_2_. Human dermal fibroblasts (HDF) were isolated from skin biopsies of healthy subjects. Briefly, the hypodermis was mechanically removed and explants were cut in 1 mm^2^ pieces. Skin fragments were then cultivated in Dulbecco’s minimal essential medium (DMEM) supplemented with 20% FBS, 1% antibiotic and 1% fungizone at 37 °C in a humidified atmosphere of 5% CO_2_. The medium was changed every two days. After 4 weeks of cultures, fibroblasts have covered all the surface of the culture dishes and were trypsined. Cells were grown in DMEM containing 10% FBS and 1% antibiotic at 37 °C in a humidified atmosphere of 5% CO_2_. Subcultures 3 to 9 were used in this study.

#### 3.6.2. Experimental Treatments

The three cell types were seeded into 96-well plates (0.32 cm^2^, 5500 cells in 200 μL medium par well). After 24 h of culture, the cells were treated in triplicate wells with different bio-produced stilbenes and synthetic resveratrol (≥99%, Sigma-Aldrich, Saint-Quentin Fallavier, France) amounts and treatment times. Compounds were dissolved in ethanol at a 5 × 10^−2^ M final concentration and stored at −20 °C. Compounds were diluted in culture media, with or without FBS, to the desired final concentration. All control and treated cells received a maximal volume of 0.1% (*v*/*v*) of ethanol.

#### 3.6.3. Cell Viability Assay

Cell viability was tested by the MTT [3-(4,5-dimethyl thiazol-2yl)-2,5-diphenyltetrazolium bromide] assay (Sigma-Aldrich) [43]. After MTT addition (0.5 mg/mL), the plates were incubated for 3 h at 37 °C in a humidified atmosphere of 5% CO_2_. At the end of the incubation period, the medium was removed and the converted dye was solubilized with dimethyl sulfoxide (DMSO). Absorbance of the converted dye was measured at a wavelength of 560 nm using a plate reader Tecan F200 Pro (Tecan, Lyon, France). The 50% inhibitory concentration (IC_50_) for each bio-produced stilbene was defined as the concentration producing 50% decrease in cell growth.

### 3.7. Statistical Analysis

The data were expressed as the mean ± SD of 3 independent experiments. Each experiment was performed in triplicate. The significance of differences was established with the Student’s *t*-test.

## 4. Conclusions

In the present study, resveratrol and various oligomeric derivatives were obtained from elicited grapevine cell suspensions (*Vitis labrusca* L.). Four stilbenes (resveratrol, ε-viniferin, pallidol and a newly characterized dimer (**6**)) were recovered in sufficient amounts to allow assessment of their biological activity on the cell growth of two human skin malignant melanoma cancer cell lines (HT-144 and SKMEL-28) and a healthy human dermal fibroblast HDF line.

It is clear from all the results obtained that resveratrol shows the best anti-cancer properties as its efficiency on cancer cell viability does not seem to be affected by the presence of fetal bovine serum. Moreover, this compound displayed a tumor-specificity. Oligomers like ε-vniferin and the dimer (**6**) have shown a high inhibiting activity of the cancer cell viability (higher than the one displayed by resveratrol), although this activity was significantly decreased in the presence of FBS. Some studies have previously evidenced a synergistic action of stilbenes used in combination compared to resveratrol alone [44,45]. Namely, a higher inhibiting activity of resveratrol extracts enriched with viniferin from *V. vinifera* cell suspensions on the viability of various cancer lines has been observed over resveratrol alone [46]. A synergistic effect between ε-vniferin, the dimer (**6**) and resveratrol used in combination could thus be envisaged in further works.

## Figures and Tables

**Table 1:** IC_50_ of bioproduced stilbenes with or without FBS in HT-144, SKMEL-28 and HDF cells at 24 h as determined by the MTT assay.

Stilbene Bioproducts	IC_50_ (µM)					
	HT-144		SKMEL-28		HDF	
	with FBS	without FBS	with FBS	without FBS	with FBS	without FBS
Trans-resveratrol	93.32 ± 7.60	> 100	> 100	> 100	> 100	> 100
Pallidol	> 100	> 100	> 100	> 100	> 100	> 100
ɛ-viniferin	82.31 ± 3.23	17.58 ± 0.09	86.62 ± 2.59	15.88 ± 0.44	91.94 ± 11.34	16.41 ± 1.19
Dimer (**6**)	> 100	24.94 ± 0.62	> 100	23.01 ± 0.30	> 100	22.21 ± 2.61

Data are expressed as IC50 values (µM) and are means ± SD of 3 independent experiments.
